# Mortality of IgA Nephropathy Patients: A Single Center Experience over 30 Years

**DOI:** 10.1371/journal.pone.0051225

**Published:** 2012-12-04

**Authors:** Hajeong Lee, Dong Ki Kim, Kook-Hwan Oh, Kwon Wook Joo, Yon Su Kim, Dong-Wan Chae, Suhnggwon Kim, Ho Jun Chin

**Affiliations:** 1 Department of Internal Medicine, Seoul National University Hospital, Seoul, Korea; 2 Department of Immunology, Seoul National University College of Medicine, Seoul, Korea; 3 Kidney Research Institute, Seoul National University Hospital, Seoul, Korea; 4 Department of Internal Medicine, Seoul National University Bundang Hospital, Seongnam, Korea; Tehran University of Medical Sciences, Iran (Republic of Islamic)

## Abstract

Research on the prognosis of IgA nephropathy (IgAN) has focused on renal survival, with little information being available on patient survival. Hence, this investigation aimed to explore long-term patient outcome in IgAN patients. Clinical and pathological characteristics at the time of renal biopsy were reviewed in 1,364 IgAN patients from 1979 to 2008. The outcomes were patient death and end stage renal disease (ESRD) progression. Overall, 71 deaths (5.3%) and 277 cases of ESRD (20.6%) occurred during 13,916 person-years. Ten-, 20-, and 30-year patient survival rates were 96.3%, 91.8%, and 82.7%, respectively. More than 50% patient deaths occurred without ESRD progression. Overall mortality was elevated by 43% from an age/sex-matched general population (GP) (standardized mortality ratio [SMR], 1.43; 95% confidence interval [CI], 1.04–1.92). Men had comparable mortality to GP (SMR, 1.22; 95% CI, 0.82–1.75), but, in women, the mortality rate was double (SMR, 2.17; 95% CI, 1.21–3.57). Patients with renal risk factors such as initial renal dysfunction (estimated glomerular filgration rate <60 ml/min per 1.73m^2^; SMR, 1.70; 95% CI, 1.13–2.46), systolic blood pressure ≥140 mmHg (SMR, 1.88; 95% CI, 1.19–2.82) or proteinuria ≥1 g/day (SMR, 1.66; 95% CI, 1.16–2.29) had an elevated mortality rate. Patients with preserved renal function, normotension, and proteinuria <1 g/day, however, had a similar mortality rate to GP. When risk stratification was performed by counting the number of major risk factors present at diagnosis, low-risk IgAN patients had a mortality rate equal to that of GP, whereas high-risk patients had a mortality rate higher than that of GP. This investigation demonstrated that overall mortality in IgAN patients was higher than that of GP. Women and patients with renal risk factors had a higher mortality than that of GP, Therefore, strategies optimized to alleviate major renal risk factors are warranted to reduce patient mortality.

## Introduction

IgA nephropathy (IgAN) is the most common form of glomerular disease worldwide, with an incidence that ranges from 20% to 40% in patients with primary glomerulonephritis [1]. The relative incidence of IgAN has increased recently, especially in Korea [2]. Despite the well-known heterogeneity of the disease and a generally slow course of disease progression, IgAN is a significant contributor to end stage renal disease (ESRD) progression [1], [3], [4]. Indeed, numerous studies have addressed the clinical [3]–[9] and pathological [3], [10]–[14] risk factors linked to the risk of progression. These include initial renal impairment [3], [5], [9], heavier or prolonged proteinuria [4]–[6], [8], hypertension [6], [8], [9], and several histological changes [3], [6], [8], [14]. However, the mortality data are not reported in most IgAN survival studies. Patient death has been considered as one part of a composite outcome [8], [12], [15] or analyzed only descriptively [16]. The mortality rate or its predictors have not been addressed in previous studies. Therefore, according to the Kidney Disease: Improving Global Outcomes (KDIGO) Clinical Practice Guidelines for glomerulonephritis (to be published), there is an assumption that IgAN patients had higher mortality than the general population (GP), and that cardiovascular morbidity and mortality increase in these patients, as in others with chronic kidney disease.

IgAN patients are usually diagnosed at a relatively young age, and most have a benign clinical course in our clinical practice. Moreover, these patients are thought to be more likely to receive transplantation because of their relatively younger age even after ESRD progression compared to their diabetic ESRD counterparts. Such clinical experiences suggest a favorable patient outcome in IgAN patients. Therefore, the rate of IgAN progression to patient death needs to be clarified, as do the clinical or pathological risk factors involved. The main purpose of this retrospective observational study was to describe the definitive patient outcome and analysis of their predictive factors, compared with renal outcome and its indicators.

**Table 1:** Baseline demographic and clinical characteristics.

		Death			ESRD		
Parameters	Total	No	Yes	*P*	No	Yes	*P*
**At the time of biopsy** (**n**)	1,364	1,276	71		1,067	277	
Age (years)	33(25–45)	32(22–44)	47(36–61)	<0.001	33(24–44)	36(28–46)	0.001
Sex (male)	682(50.0)	632(49.5)	43(60.6)	0.087	513(48.1)	161(58.1)	0.003
SBP (mmHg)	120(110–138)	120(110–130)	140(124–150)	<0.001	120(110–130)	130(120–150)	<0.001
Co-morbidity							
Diabetes	25(2.0)	22(1.9)	3(5.0)	0.120	18(1.9)	7(2.7)	0.037
Cancer	10(0.8)	4(0.3)	6(10.0)	<0.001	7(0.7)	3(1.2)	0.448
Hypertension	484(38.7)	442(37.2)	42(67.7)	<0.001	319(32.4)	165(62.3)	<0.001
Clinical manifestations							
Edema	314(24.1)	288(23.2)	26(40.0)	0.004	210(20.3)	104(38.4)	<0.001
Gross hematuria	438(33.2)	417(33.3)	21(31.3)	0.791	370(35.4)	70(25.5)	0.002
AUA	457(35.0)	442(35.6)	15(22.7)	0.034	386(37.3)	71(26.0)	<0.001
Laboratory tests							
Hemoglobin (g/dL)	13.3(11.8–14.6)	13.3(11.9–14.6)	11.7(9.4–13.9)	<0.001	13.5(12.1–14.7)	12.1(10.5–13.9)	<0.001
Albumin (g/dL)	3.9(3.5–4.2)	3.9(3.5–4.2)	3.3(2.7–3.9)	<0.001	3.9(3.6–4.2)	3.6(3.1–4.0)	<0.001
Cholesterol (mg/dL)	186(158–220)	186(158–219)	209(154–239)	0.125	184(157–216)	203(166–233)	<0.001
Creatinine (mg/dL)	1.10(1.10–1.50)	1.10(0.90–1.40)	1.50(1.20–2.15)	<0.001	1.10(0.90–1.30)	1.70(1.20–2.40)	<0.001
eGFR (mL/min/1.73m^2^)	67.6(27.6)	68.7(27.4)	48.3(25.4)	<0.001	73.5(25.2)	45.7(24.9)	<0.001
24hour proteinuria (g/day)	1.30(0.56–2.50)	1.22(0.54–2.36)	2.62(1.60–5.44)	<0.001	1.11(0.50–2.12)	2.11(1.03–3.46)	<0.001
**During follow-up** (**n**)	1223	1163	60		965	258	
Development of cancer	47(3.8)	35(3.0)	12(20.0)	<0.001	30(3.1)	17(6.6)	0.016
Development of diabetes	73(6.0)	66(5.7)	7(9.6)	0.084	50(5.2)	23(8.9)	0.037
Medical treatment (n)	1050	1009	41		842	208	
Antiplatelet agents	695(64.8)	322(31.3)	6(14.0)	0.017	265(30.9)	63(29.6)	0.740
Statin	146(13.6)	142(13.8)	4(9.3)	0.501	117(13.6)	29(13.6)	1.000
RAS blockade	328(30.6)	675(65.6)	20(46.5)	0.014	557(64.9)	137(64.3)	0.873
Immunosuppressant	137(12.7)	124(12.0)	13(30.2)	0.002	103(12.0)	34(15.9)	0.137

All continuous variables are shown as mean (SD) for normal distributions, or median (interquartile range) for non-parametric variables. Categorical variables were frequency per observation (N (%)). Baseline characteristics for patients who progressed to the primary outcome were compared with those who did not using χ^2^ test for dichotomous variables, and student t-test for parametric continuous variables.

Abbreviations: ESRD, end stage renal disease; BMI, body mass index; AUA, asymptomatic urinary abnormalities; SBP, systolic blood pressure; DBP, diastolic blood pressure; eGFR, estimated glomerular filtration rate; TA, tubular atrophy; RAS, renin-angiotensin system.

## Materials and Methods

### Ethics statement

This investigation was approved by the institutional review board in Seoul National University Hospital and was in accordance with the principle of the Helsinki Declaration II (H-1010-055-336). As the study was retrospective in design and did not include any interventions, informed consent was waived.

### Study subjects

From 1979 to 2008, a kidney biopsy registry was constructed using 4,998 kidney needle biopsy cases among patients aged ≥15 years at the Seoul National University Hospital. Allograft biopsy cases were excluded from this cohort. Among the retrospective cohort, a primary diagnosis of IgAN was made in 1,379 patients. Fifteen of these patients who had less than 5 glomeruli in their biopsy specimen had insufficient information for diagnosis and were excluded from this study [11], [17]. The diagnosis was based on immunofluorescence microscopy showing mesangial IgA deposition as the predominant or co-dominant immunoglobulin, and on the lack of clinical or laboratory evidence of systemic lupus erythematosus, Henoch-Schonlein nephrtis, or liver cirrhosis. In case of lupus nephritis, only the patients with clinical suspicion for lupus nephritis were further tested for lupus autoantibodies.

**Table 2:** Pathologic changes of study population.

		Death			ESRD		
Parameters	Total (n = 1,270)	No(n = 1190)	Yes(n = 67)	*P*	No(n = 858)	Yes(n = 219)	*P*
Number of glomerulus	64(21–55)	35(22–56)	26(18–43)	0.007	38(23–58)	26(17–40)	<0.001
Global sclerosis (%)	14.8(3.7–34.9)	14.3(3.4–34.2)	25(5.6–46.2)	0.020	11.3(2.4–27.2)	37.5(19.2–59.3)	<0.001
Segmental sclerosis (%)	6.8(0–14.3)	6.9(0–14.3)	3.3(0–16.7)	0.193	6.1(0–13.0)	11.2(3.4–19.1)	<0.001
Crescent (yes)	268(21.3)	246(20.7)	22(32.8)	0.022	210(21.2)	58(21.8)	0.866
TA/Interstitial fibrosis				0.004			<0.001
None	119(9.6)	114(9.7)	5(7.9)		106(10.8)	13(5.0)	
Mild	517(41.8)	498(42.4)	19(30.2)		472(48.3)	45(17.2)	
Moderate	375(30.3)	358(30.5)	17(27.0)		291(23.5)	84(32.1)	
Severe	228(18.3)	205(17.4)	22(34.9)		108(11.1)	120(45.8)	
Interstitial inflammation				0.014			<0.001
None	163(13.2)	155(13.2)	7(11.1)		133(13.6)	30(11.5)	
Mild	485(39.1)	467(39.7)	18(26.6)		447(45.8)	38(14.5)	
Moderate	380(30.7)	362(30.8)	18(26.6)		293(30.0)	87(22.9)	
Severe	211(17.0)	191(16.3)	20(31.7)		104(10.6)	107(40.8)	
Vascular change				0.042			<0.001
None	769(62.0)	736(62.7)	32(50.0)		657(67.2)	112(43.1)	
Hyalinosis	215(17.4)	199(17.0)	16(25.0)		148(15.1)	66(25.4)	
Atherosclerotic change	255(20.6)	239(20.3)	16(25.0)		173(17.7)	82(31.6)	
WHO pathologic grade (n)	1077	1027	50		858	219	
I	31(2.9)	31(3.0)	0(0.0)		29(3.4)	2(0.9)	
II	272(25.3)	267(26.0)	5(10.0)		265(30.9)	7(3.2)	
III	464(43.1)	446(43.5)	18(36.0)		398(46.4)	66(30.1)	
IV	191(17.8)	178(17.3)	13(26.0)		124(14.5)	67(30.6)	
V	118(11.0)	104(10.1)	14(11.9)		42(4.9)	77(35.2)	

All continuous variables are shown as mean (SD) for normal distributions, or median (interquartile range) for non-parametric variables. Categorical variables were frequency per observation (N (%)). Pathological characteristics for patients who progressed to the primary outcome were compared with those who did not using χ^2^ test for dichotomous variables, and student t-test for parametric continuous variables. Abbreviations: ESRD, end stage renal disease; TA, tubular atrophy;

### Clinical data

Baseline demographic and clinical characteristics were obtained from a review of the medical records at the time of biopsy. Demographic and clinical parameters including age, sex, blood pressure, blood chemistry analysis and 24-h urine protein were obtained. Information about co-morbidities was also collected. Hypertension was defined as a reported history of hypertension, a systolic blood pressure (SBP) ≥140 mmHg, or a diastolic blood pressure ≥90 mmHg. Diabetes mellitus was defined as a reported history of diabetes or as the active use of an oral hypoglycemic agent or insulin. Anemia was defined as a hemoglobin level <13 g/dL for men and <12 g/dL for women. The estimated glomerular filtration rate (eGFR) was calculated by the modified modification of diet in renal disease equation after measuring serum creatinine. Data on medication were collected if any of the following was started within 6 months of renal biopsy and was prescribed for more than 3 months: renin-angiotensin system blockades, including any kind of angiotensin-converting enzyme inhibitor and angiotensin receptor blocker; any kind of glucocorticoid; statins; and antiplatelet agents such as aspirin or clopidogrel.

**Figure 1:**
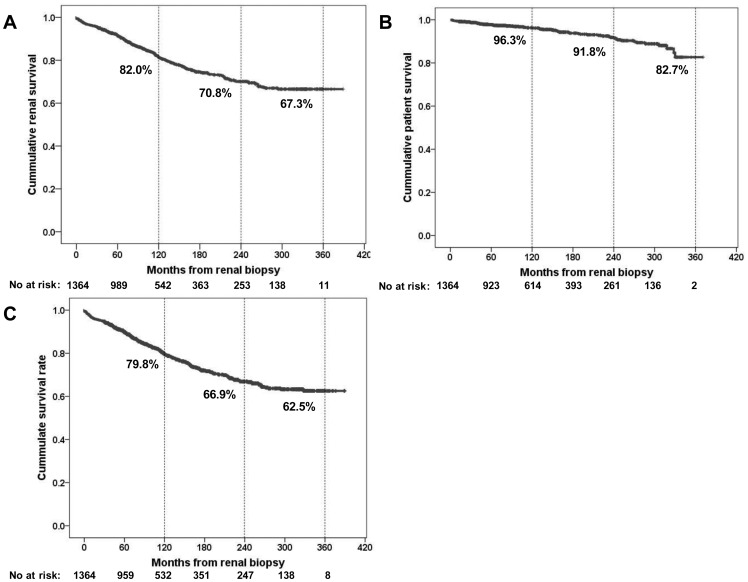
Cumulative renal and patient survival after renal biopsy. The primary endpoint was renal (A) and patient survival (B) and composite outcome (C). The numbers of patients remaining at 60, 120, 180, 240, 300, and 360 months of follow-up are shown at the bottom. ESRD, end stage renal disease.

To evaluate histopathological change, 2 pathologists reviewed the renal biopsy slides. In the glomerular area, the numbers of glomeruli, proportions of global sclerosis, segmental sclerosis, and crescent lesion were calculated. The percentages of glomeruli with these lesions were deduced and categorized. In the tubulointerstitial area, tubular atrophy and interstitial fibrosis, interstitial inflammatory cell infiltration, and vascular change were graded. The histopathological grades were also analyzed using the WHO grading system for IgAN.

### Outcome measurement

The outcomes were the death from any cause and ESRD progression (permanent hemodialysis, peritoneal dialysis or renal transplantation) after renal biopsy. Data on mortality and cause of death were obtained from the Korean National Statistical Office (KNSO), and ESRD data were collected from the Korea ESRD registry [18]. We combined all these data according to the unique identification number held by all Koreans. In addition, the medical records were searched retrospectively to obtain additional information related to the primary outcome and the recent renal function of the patients. It was assumed that patients who had no follow-up with our institution and no follow-up creatinine values, and who did not undergo any renal replacement therapy or a reported death did not meet the primary endpoint at the time the database closed.

**Figure 2:**
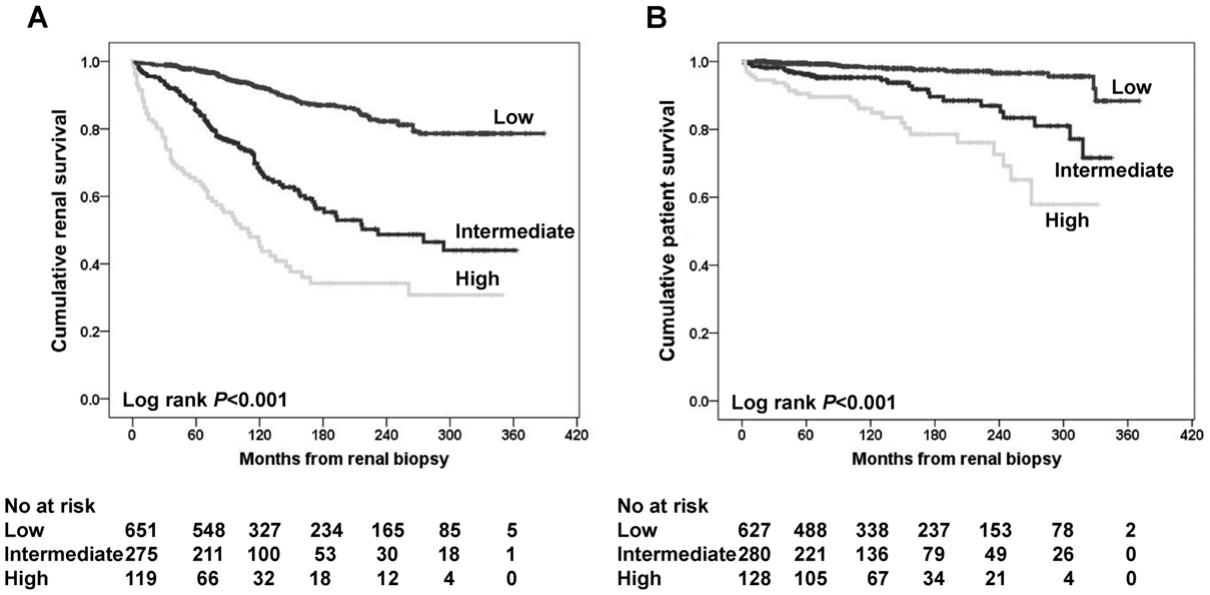
Cumulative renal (**A**) **and patient survival** (**B**) **according to the risk stratification.** The primary outcome is patient survival. The numbers of patients remaining at 60, 120, 180, 240, 300, and 360 months of follow-up are shown at the bottom.

**Table 3:** Univariate and multivariate time dependent cox regression analyses for patient death and renal death.

		Univariate analysis				Multivariate analysis			
		Wald	HR	95% CI	P	Wald	HR	95% CI	P
**ESRD**	eGFR ≥90(mL/min/1.73m^2^)	Ref.	Ref.	Ref.		Ref.	Ref.	Ref.	
	60–90	14.403	4.151	1.990–8.658	<0.001	5.024	2.395	1.116–5.141	0.025
	30–60	48.996	12.988	6.335–26.627	<0.001	27.808	7.330	3.496–15.368	<0.001
	15–30	91.860	40.496	18.998–86.322	<0.001	35.737	12.828	5.557–29.612	<0.001
	<15	122.268	84.995	38.673–186.804	<0.001	69.844	41.724	17.393–100.092	<0.001
	Hypertension	57.262	2.693	2.084–3.481	<0.001	9.667	1.698	1.216–2.370	0.002
	Segmental sclerosis ≥20%	38.746	2.480	1.863–3.301	<0.001	8.063	1.674	1.173–2.389	0.005
	Gross hematuria	31.694	0.435	0.326–0.582	<0.001	5.753	0.613	0.411–0.914	0.016
	Albumin <3.5 g/dL	63.312	2.741	2.138–3.514	<0.001	4.416	1.429	1.024–1.993	0.036
**Death**	Age <40 years	Ref.	Ref.	Ref.		Ref.	Ref.	Ref.	
	40–59	20.440	3.718	2.104–6.571	<0.001	4.626	2.229	1.074–4.626	0.031
	≥60	101.088	24.493	13.130–45.691	<0.001	49.267	15.627	7.253–33.670	<0.001
	SBP ≥140 mmHg	28.157	3.730	2.294–6.065	<0.001	9.121	2.484	1.376–4.482	0.003
	Albumin <3.5 g/dL	39.264	4.778	2.930–7.794	<0.001	8.481	2.470	1.344–4.539	0.003
	Cancer	28.999	5.745	3.040–10.855	<0.001	3.943	2.224	1.010–4.894	0.047
**Composite**	Age <40 years	Ref.	Ref.	Ref.		Ref.	Ref.	Ref.	
	40–59	16.913	1.695	1.318–2.180	<0.001	1.520	0.672	0.358–1.264	0.218
	≥60	60.809	4.582	3.125–6.718	<0.001	14.317	5.351	2.244–12.757	<0.001
	Cancer	23.350	2.760	1.828–4.166	<0.001	13.545	2.882	1.640–5.064	<0.001
	eGFR ≥90 (mL/min/1.73m^2^)	Ref.	Ref.	Ref.		Ref.	Ref.	Ref.	
	60–90	15.181	3.523	1.870–6.639	<0.001	1.306	1.518	0.742–3.107	0.253
	30–60	58.847	11.161	6.026–20.672	<0.001	13.687	3.940	1.906–8.147	<0.001
	15–30	111.663	34.982	18.092–67.639	<0.001	18.046	6.246	2.682–14.542	<0.001
	<15	118.059	46.326	23.192–92.536	<0.001	23.710	9.675	3.881–24.120	<0.001
	WHO grade I–III	Ref.	Ref.	Ref.		Ref.	Ref.	Ref.	
	IV	83.574	4.433	3.222–6.101	<0.001	7.818	1.847	1.201–2.840	0.005
	V	231.786	11.790	8.581–16.197	<0.001	38.291	4.625	2.847–7.511	<0.001
	SBP ≥140 mmHg	62.387	2.543	2.017–3.206	<0.001	7.136	1.578	1.129–2.206	0.008

Multivariate time-dependent cox regression analysis for patient-death was included age, sex, clinical manifestations of edema/gross hematuria, co-morbidities of hypertension/cancer, BMI, GFR, anemia, albumin <3.5g/dL, SBP ≥140 mmHg, DBP ≥90 mmHg, proteinuria ≥1 g/day, and pathologic change of global sclerosis, presence of crescent, interstitial inflammation and tubular atrophy/interstitial fibrosis. Sex and global sclerosis did not meet proportional hazards assumption for Cox model. Global sclerosis interacted with GFR and interstitial inflammatory cell infiltration. BMI interacted with age. Therefore such interactions were considered in this model.

Multivariate time-dependent cox regression analysis for renal-death was included age, sex, clinical manifestations of edema/gross hematuria, co-morbidities of diabetes/hypertension/cancer, GFR, anemia, albumin <3.5g/dL, SBP ≥140 mmHg, DBP ≥90 mmHg, proteinuria ≥1 g/day, pathologic change of segmental sclerosis, and treatment history with statin and renin-angiotensin system blockades. Sex and age were considered changes of proportional hazard according to time progression. Global sclerosis and other tubulointerstial changes were excluded in the final model because of severe interaction with GFR.

Abbreviations: SBP, systolic blood pressure; ESRD, end-stage renal disease; eGFR, estimated glomerular filtration rate; HR, Hazard ratio; CI, confidence interval.

### Statistical analysis

The data are presented as frequencies and percentages for categorical variables. Continuous variables with normal distribution are indicated as mean ± SD, while those without normal distribution are shown as median and interquartile range (IQR). Comparisons between the outcome group and other groups were performed using the χ^2^ test for dichotomous variables, Student *t*-test for parametric continuous variables, and Mann-Whitney test for non-parametric continuous variables.

**Table 4:** Causes of death.

Causes of death (N)	Death before ESRD (n = 39)	Death after ESRD (n = 31)	Total
Renal disease	2	11	13
Cardiovascular disease	5	5	10
Cancer	12	1	13
Infection	6	4	10
Traffic accident or injury	3	1	4
Miscellaneous	2	3	5
Unknown	9	6	15

Abbreviations: ESRD, end-stage renal diseas.

Survival rates for ESRD, death, and composite outcome were analyzed using the Kaplan-Meier method. Survival differences were tested by the log-rank procedure. Cox proportional hazards models were used for prognostic factor assessment. Proportional hazards assumption for Cox models were tested by using log-minus-log plots. Variables that failed to satisfy the proportional hazards assumptions were analyzed by using time-dependent Cox regression analysis. Variables that showed a significant association (*P*<0.10) in the univariate analysis or that were of considerable theoretical relevance were retained as potential predictors in the multivariate model. In the forward conditional multivariate models, the orders of variable selection and the values of Wald statistics helped to determine the rankings of the risk factors.

**Figure 3:**
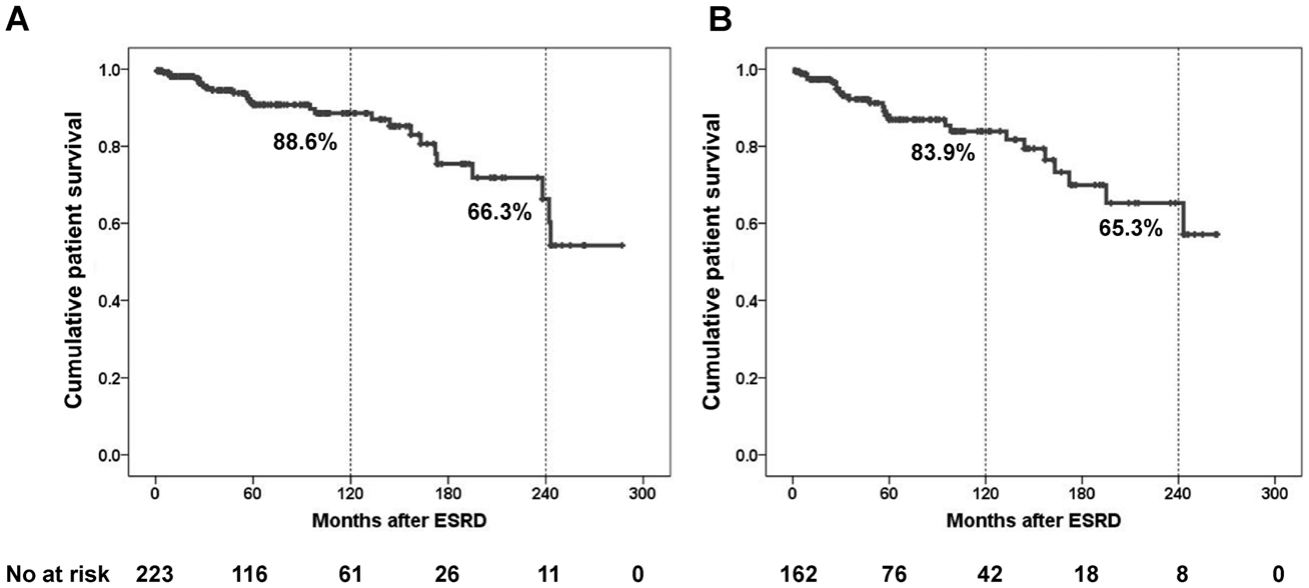
Cumulative patient survival after ESRD progression according to the all ESRD patients (**A**) **and excluding transplantation recipients** (**B**)**.** The primary outcome is patient survival. The numbers of patients remaining at 60, 120, 180, 240, 300, and 360 months of follow-up are shown at the bottom. ESRD, end stage renal disease.

From the results of Cox-regression analyses, we identified 3 major risk factors: SBP ≥140 mmHg, proteinuria ≥1 g/day, and baseline renal insufficiency with eGFR <60 ml/min per 1.73m^2^. These major risk factors were simplified as a sum of present risk factors as follows: low-risk group for none or one of the risk factors; intermediate-risk group for any 2 of the risk factors, and high-risk group for all their simultaneous presence. Patient survival and renal survival analyses were also performed according to the risk stratification.

**Table 5:** Causes of death according to use of immunosuppressive agents.

	Steroid IV	Steroid PO	Cyclophosphamide	Calcineurin inhibitor	Mycophenolate
No of prescription	25	130	36	9	9
Cumulative dose	1187(450–2437)	3115(1519–4308)	6487(4087–10050)	1833	107000
Duration	3(1.8–6.0)	122(75–168)	80(48–123)	107	111
No of death	3	12	6	2	1
Cause of death					
Renal disease	0	0	1	0	0
Cardiovascular disease	0	1	1	0	0
Cancer	0	2	0	0	0
Infection	2	5	2	2	1
Unknown	1	4	2	0	0

Abbreviations: IV, intravenous; PO per oral;

To clarify the mortality rate associated with IgAN, the standardized mortality ratio (SMR) was calculated as the ratio between the observed and the expected number of deaths. The expected number of deaths was calculated by person-year methods as follows: (1) The sum of annual observed person-years was calculated during the observation period (1992–2008). (2) The expected number of deaths was calculated by multiplying the sum of annual observed person-years by sex-adjusted national mortality data in 5-year calendar periods and 5-year age groups. (3) The sum of annual expected number of deaths was calculated. Information about the annual mortality rates of the general Korean population was collected from the KNSO. Because national mortality statistics were available from 1992, SMR was calculated in the patients who had a renal biopsy after 1992. An SMR >1.0 was considered to be an excess mortality. To calculate the 95% confidence intervals (CIs) for the SMR of each group, the Poisson-distributed number of observed cases was assumed [19]. Two-sided *P* values are reported, with the level of statistical significance set at 0.05. The SPSS Statistics (version 19.0, Chicago, IL, USA) package was used for statistical analysis.

**Table 6:** Standardized mortality ratios (SMRs) in overall and subpopulation of IgAN patients.

		N	Initial age	Final age	Person-year	Observed	Expected	SMR(95% CI)
**Overall**		1009	36.8±13.7	45.0±14.0	8134.2	44	30.7	1.43(1.04–1.92)
eGFR	≥60	606	32.9±12.2	41.3±13.2	5077.4	15	13.8	1.08(0.61–1.79)
	<60	374	43.4±13.6	50.9±13.2	2825.6	28	16.5	1.70(1.13–2.46)
Proteinuria	<1 g/day	341	33.8±13.1	41.0±13.3	2445.5	6	6.2	0.97(0.36–2.12)
	≥1 g/day	572	39.1±13.7	47.7±13.7	4941.5	36	21.7	1.66(1.16–2.29)
SBP	<140	768	35.2±13.1	42.8±13.4	5911.3	18	18.3	0.98(0.58–1.56)
	≥140	230	42.2±14.1	51.4±13.9	2125.2	23	12.3	1.88(1.19–2.82)
**Men**		495	36.1±14.7	45.0±15.0	4403.6	29	23.8	1.22(0.82–1.75)
eGFR	≥60	309	31.6±12.9	40.7±13.6	2827.9	10	10.2	0.98(0.47–1.81)
	<60	173	44.6±14.0	53.1±13.5	1482.8	18	13.4	1.34(0.80–2.13)
Proteinuria	<1 g/day	143	31.5±13.7	40.1±14.0	1225.5	3	1.9	1.57(0.32–4.58)
	≥1 g/day	307	38.7±14.4	47.8±14.3	2791.7	12	4.6	2.60(1.35–4.55)
SBP	<140	356	34.4±14.3	43.0±14.5	3092.0	12	14.1	0.85(0.44–1.49)
	≥140	135	40.6±14.9	50.2±14.6	1299.1	15	9.7	1.55(0.87–2.56)
**Women**		514	37.5±12.6	45.0±13.0	3730.6	15	6.9	2.17(1.21–3.57)
eGFR	≥60	297	34.4±11.3	41.9±12.7	2249.5	5	3.7	1.36(0.44–3.18)
	<60	201	42.4±13.2	49.0±12.8	1342.8	10	3.1	3.24(1.55–5.95)
Proteinuria	<1 g/day	198	35.5±12.4	41.6±2.9	1220.0	3	4.2	0.71(0.15–2.07)
	≥1 g/day	265	39.4±12.7	47.5±13.0	2149.8	24	17.1	1.40(0.90–2.08)
SBP	<140	412	35.9±12.0	42.7±12.4	2819.3	6	4.2	1.43(0.53–3.12)
	≥140	95	44.5±12.5	53.1±12.7	826.1	8	2.6	3.09(1.34–6.10)

Abbreviations: SMR, standardized mortality ratio; CI, confidence interval; eGFR, estimated glomerular filtration rate; SBP, systolic blood pressure.

## Results

### Baseline characteristics according to outcome development

Overall, 1,364 patients were included in the final analysis. Initial demographic and clinical data are listed in Table 1. The median age at the time of biopsy was 33 years (IQR, 25–45). The proportion of men and women was equal, although age distribution according to sex was quite different. Median age was lower in men (31 years; IQR, 22–45) than in women (35 years; IQR, 27–45). Gross hematuria was present in 33.2% of patients. The mean eGFR was 67.6 ml/min per 1.73m^2^ and proteinuria was 1.3 g/day. In all, 137 patients were treated with immunosuppressive agents, of which 25 were treated with intravenous steroids; 130 with oral steroids; 36 with oral cyclophosphamide; 9 with cyclosporine; and 9 with mycophenolate mofetil.

**Figure 4:**
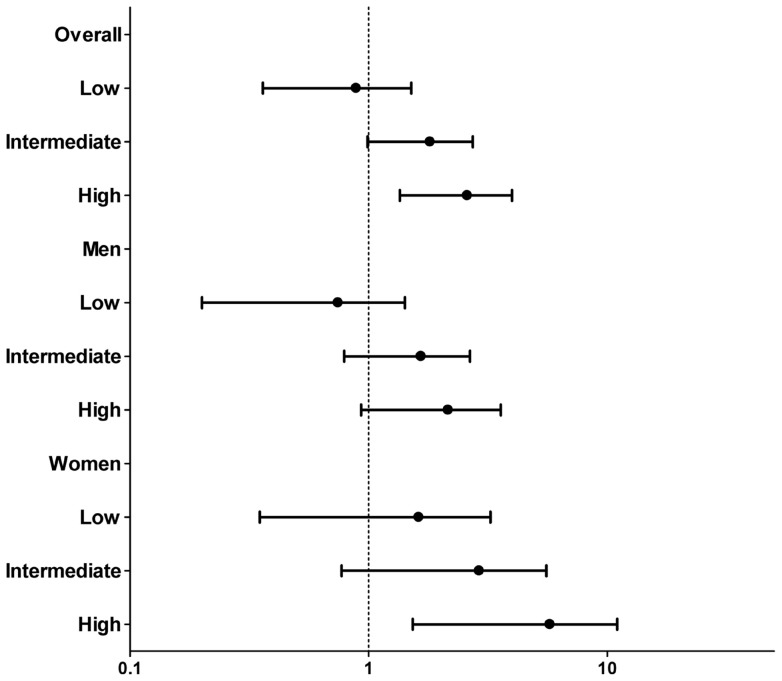
Standardized mortality ratio (**SMR**) **according to risk stratification.** The x-axis represents SMR with 95% confidence interval in log scale. The y-axis represents overall and gender subgroup of IgAN patients.

The patients who died were significantly older, and had higher blood pressure, more nephrotic features and more depressed renal function at the time of biopsy than did the survivors. A higher proportion of the patients who died was managed with immunosuppressive agents before death. Patients with ESRD progression were slightly older and included a higher proportion of men than did the non-ESRD group. Nephrotic features, higher blood pressure, lower hemoglobin level and initial renal dysfunction were significantly higher in patients with renal progression. Table 2 summarizes the pathological data of the study population. The pathological changes were more severe in both glomerular and tubulointerstitial areas in ESRD patients and in those who died.

Figure 1 compares the overall renal and patient survival rates. In cases of renal survival, 277 (20.6%) patients advanced to renal death. Ten, 20- and 30-year renal survival rates were 82.0%, 70.8% and 67.3%, respectively during a median observation period of 96 (IQR, 56–187) months with 14,495 person-years. The median time to ESRD was 71 (IQR, 32–123) months. Seventy-one (5.3%) patients died during the median observation period of 100 (IQR, 51–210) months, with 13,916 person-years. The median time to death was 101 (IQR, 38–189) months. Ten, 20-, and 30-year patient survival rates were 96.3%, 91.8%, and 82.7%, respectively. For composite outcome, the median observation period was 96 months (IQR, 57–188), with 14,588 person-years. The median time to event was 69 (IQR, 31–165) months. Three-hundred and sixteen (23.2%) patients reached the composite outcome during the long-term follow-up. Ten, 20-, and 30-year patient survival rates were 79.8%, 66.9%, and 62.5%, respectively.

### Comparisons of predictors according to renal and patient outcome

Risk factor assessment was performed according to ESRD, death, and composite outcome, respectively, In the univariate analysis, renal survival was predominantly affected gradually by eGFR stage in the univariate analysis, even from an eGFR <90 ml/min per 1.73m^2^. SBP ≥140 mmHg, proteinuria ≥1 g/day, hypoalbuminemia and edema were the next important determinants of ESRD progression. For death, age was a predominant risk factor even from the age of 40 years. Combined malignancy; SBP ≥140 mmHg; eGFR <60 ml/min per 1.73m^2^; nephrotic features such as edema, proteinuria, and hypoalbuminemia; and anemia were the next significant predictors of patient death. None of the medical treatment had any demonstrable effect on mortality. In addition, patient survival was influenced by glomerular changes such as global sclerosis, segmental sclerosis, and the presence of any crescent formation. Severe degrees of tubulointerstitial changes were also associated with poor patient survival. For composite outcome, the significant predictors were similar to those of ESRD.

The results of the multivariate analysis for the indicators of ESRD and patient death are summarized in Table 3. In the case of renal survival, initial renal function was the most important determinant, followed, as expected, by hypertension. Segmental sclerosis and hypoalbuminemia remained as significant predictors of renal progression. Gross hematuria was associated with a favorable renal outcome. For patient outcome, advanced age, SBP ≥140 mmHg, hypoalbuminemia and combined malignancy were identified as independent determinants. Interestingly, predictors of patient and renal death appeared to be similar, with hypoalbuminemia and hypertension (or SBP ≥140 mmHg) being common risk factors for both outcomes. Moreover, determinants of the composite outcome seemed to be the combination of the predictors of mortality and ESRD progression. When the simplified risk stratification was applied, it predicted both renal and patient outcome in IgAN patients well (Figure 2).

### Subgroup analysis for patient death

Among the 71 deaths, 39(55.7%) patients died before ESRD progression. These 39 patients were significantly older (median age 49 [IQR, 38–64] vs.61 [IQR, 53–68] years, *P* = 0.049) and showed relatively conserved renal function (median eGFR 55.8 [IQR, 39.1–71.5] vs. 37.0 [IQR, 18.3–53.7] ml/min per 1.73m^2^, *P* = 0.004) compared to the rest. However, other clinical factors were not different between the 2 groups). Table 4 summarizes the causes of death. The causes of death in the patients who died before advancing to ESRD progression were malignancies (12 patients, 30.8%), cardiovascular diseases (5 patients, 12.8%), and infection (6 patients, 15.8%). Death from ESRD and dialysis-related complications occurred in only 2 patients. In contrast, the deaths after ESRD progression were caused by renal disease (11 patients, 35.5%), and cardiovascular disease (5 patients, 16.1%), with only one case of cancer mortality.

As expected, ESRD progression was a significant predictor of mortality (hazard ratio, 2.593; 95% CI, 1.609–4.177; *P* <0.001). After ESRD progression, 25.3% patients received renal transplantation. Among the patients who received transplantation, only 3 patients died, 1 from infection and the other 2 after allograft failure and resumption of dialysis. All of them were dead by 12 years after ESRD progression. As a result, although the 10-year survival rate after ESRD was higher in all ESRD patients including transplantation recipients, than in those excluding transplantation recipients, the 20-year survival rate of ESRD was similar in both groups at about 65% (Figure 3).

Thirteen patients who were managed by immunosuppressive agents had died. Among them, 5 patients died from infection and two patients died from malignancy. Three patients whose cause of death was infection had underlying cancer (Table 5). There was no relationship between the cumulative dose of immunosuppressive agents and any death or infection-associated death (data was not shown).

### Standardized mortality ratio in IgAN

Table 6 summarizes the overall and sex-specific SMR results. The overall relative mortality rate of IgAN patients was significantly higher by 43% than that of age/sex-matched GP (SMR, 1.43; 95% CI, 1.04–1.92). Interestingly, the mortality rate was different according to subgroups. An excess mortality rate was found in women (SMR, 2.17; 95% CI, 1.21–3.57), but not in men (SMR, 1.22; 95% CI, 0.82–1.75) with IgAN. Moreover, patients with lower eGFR (<60 ml/min per1.73m^2^; SMR, 1.70; 95% CI, 1.13–2.46), higher proteinuria (≥1 g/day; SMR, 1.66; 95% CI, 1.16–2.29), or higher SBP (≥140 mmHg; SMR, 1.88; 95% CI, 1.19–2.82) had an elevated mortality rate compared with their age/sex-matched GP, whereas patients without such risk factors had a similar mortality rate.

When the SMR was further classified by the simplified risk stratification, IgAN patients in the low-risk group had a similar mortality rate to the GP. In the intermediate-risk group, IgAN patients’ mortality appeared higher than that in the GP, although insignificant. However, in the high-risk group, IgAN patients’ mortality was significantly higher than that in the GP. Interestingly, this relationship was different according to sex. Thus, risk stratification did not have any influence on the SMR in men, but SMR in women was affected significantly (Figure 4).

## Discussion

Until recently, investigations on IgAN have focused on renal prognosis because it is the most common primary glomerulonephritis and a significant contributor to the development of ESRD, despite the slow rate of progression [1], [20]. However, there has been no information about death, which is more definitive outcome than ESRD. In this investigation, we demonstrated that the 30-year mortality of IgAN patients was 82.7%. Moreover, we showed that mortality of IgAN patients was higher than that of the age/sex-matched GP by 43%. To the best of our knowledge, this is the first study to investigate patient survival and its predictive factors as distinguished from renal survival in IgAN patients.

The most notable finding in our survival analyses is that although the overall relative mortality of IgAN, expressed by SMR, was shown to be higher than that of the GP, the absolute mortality rate was not very high when considering the significant renal progression to ESRD. More than half of the deaths occurred even before ESRD progression and the most common cause of death was malignancy. In particular, the patients who survived and progressed to ESRD had a better survival rate than the general dialysis patients did. According to the Korean ESRD registry, the 10-year survival rate of the overall dialysis patients was about 45%, and the 10-year survival rate of the non-diabetic dialysis patients within this group was 58.5% [21]. In the 2011 US Renal Data System data, survival over the first 5 years of therapy was only 45% in the patients with glomerulonephritis [22]. Compared with the above data, the ESRD patients with IgAN in this investigation have a favorable survival rate. The relatively young age of the ESRD population in this study may have explained the favorable survival. In this study, the mean age of the 277 patients who progressed to ESRD was 45 years, which was lower than the mean age (52.1 years) of the 5,550 glomerulonephritis-induced ESRD patients in the Korean ESRD registry [21]. With regard to transplantation, this may have contributed to the improved survival in the ESRD patients with IgAN for 10-year survival rate, but not the 20-year survival rate. To clarify the precise mechanisms of the fair survival rate for IgAN patients with ESRD, further well-designed investigations are needed.

Interestingly, the survival patterns differed according to the sex, renal function, proteinuria and blood pressure. Patients who were male, with preserved renal function, normotension, and proteinuria <1 g/day had a similar mortality rate compared with the GP, whereas female patients, or patients with renal dysfunction, higher blood pressure and proteinuria ≥1 g/day had significantly higher mortality, compared with the GP. The results for the 3 subgroups (renal dysfunction, higher blood pressure, and proteinuria ≥1 g/day) were similar, although the difference according to sex was unexpected result. In the regression analysis, a sex difference was not detected even in the univariate analysis. However, men showed a comparable survival rate, whereas women tended to have a higher mortality rate compared with the age-matched Korean GP, although the actual number of patients who died was higher in men. On average, men had a 20% higher death rate than did women. The difference was increased up to 3-fold in their 40s and 50s compared with Korean GP (http://kostat.go.kr). This difference may cause the discordance between the relative and absolute mortality in IgAN patients. The precise mechanisms of the sex difference in relative mortality remain to be defined.

The survival pattern of IgAN patients described in this analysis suggests the need for some changes in clinical practice. Despite the high prevalence of IgAN, adequate and specific treatment intervention in the early stage of IgAN remains controversial. Therefore, both clinicians and patients tend to hesitate before following an active therapeutic approach to individual IgAN patients, even at high risk for progression. However, the decreased age at diagnosis, reduced cardiovascular risk factors and increased average life expectancies have allowed more IgAN patients to live with ESRD state for a relatively longer duration, compared with other causes of ESRD including diabetes. ESRD treatment clearly presents a considerable burden to both society and the patients [22]. The cost per person-year of ESRD was the highest and that of transplantation was the fourth highest among the 500 common disease categories in Korea. Moreover, expenditure per person-year for ESRD has increased continuously up to US$12,639 per patient per year in 2010 (http://www.nhic.or.kr). Thus, the fair survival rate of IgAN patients also implies a substantial social cost for ESRD. Therefore, optimal treatment guidelines, including both active and supportive care need to be developed for IgAN patients according to their renal risk stratification in order to reduce renal progression.

While the present study suggested some novel findings, there are several limitations. First, this investigation was a retrospective observational study in a single center. During the long follow-up period, the general medical status and/or treatment strategy of the patients changed over time. However, we could not reflect such changes, and only took initial medical treatments into account. For this reason, the incompleteness of the data and the possibility of residual confounding cannot be excluded. For example, information about patients who received tonsillectomy was not available in our analyses. A validation study in an external cohort or a prospective multicenter study may be necessary to overcome our study limitations. The inability to elucidate reproducibility in pathological diagnosis is another weak point of our investigation.

In conclusion, we have demonstrated that the absolute 30-year survival of IgAN was 82.7%. Moreover, the relative mortality was higher than that of the age/sex-matched GP by 43%. Especially, relative mortality was higher in a subgroup with well-known renal risk factors and in women. However, patients without renal risk factors survived similarly to the GP. Considering the socioeconomic burdens of ESRD, more meticulous and active management to mitigate renal progression is warranted in IgAN patients.
